# An MEM-DMD-Enabled Ghost Imaging System Enhanced by a Hybrid CNN-GAN for High-Resolution Imaging Under Scattering Media

**DOI:** 10.3390/mi17050598

**Published:** 2026-05-14

**Authors:** Zeenat Akhter, Rehmat Iqbal, Giedrius Janusas, Sigita Urbaite, Arvydas Palevicius

**Affiliations:** 1 Department of Mechanical Engineering, Faculty of Mechanical Engineering and Design, Kaunas University of Technology, Studentų 56, LT-51424 Kaunas, Lithuania; giedrius.janusas@ktu.lt (G.J.); sigita.urbaite@ktu.lt (S.U.); arvydas.palevicius@ktu.lt (A.P.); 2 School of Optics and Photonics, Beijing Institute of Technology, Beijing 100081, China; rehmat_iqball@outlook.com

**Keywords:** ghost imaging, MEMS, digital micromirror device, adaptive illumination patterns, CNN–GAN

## Abstract

This paper presents a Micro-Electro-Mechanical Systems digital micromirror device (MEMS-DMD)-enabled ghost imaging (GI) framework for high-resolution imaging under scattering conditions. Unlike conventional ghost imaging systems that rely on fixed illumination patterns, the proposed approach exploits the high-speed programmability of a DMD to implement adaptive illumination strategies, enabling dynamic selection of informative patterns during data acquisition. This hardware-enabled pattern selection strategy improves sampling efficiency and reconstruction stability under the modeled fog conditions considered here. A hybrid convolutional neural network–generative adversarial network (CNN–GAN) model is employed as an inversion tool to reconstruct high-quality images from compressed bucket measurements. The proposed system achieves substantial improvements in reconstruction quality, with 23–40% gains in PSNR and 18–26% in SSIM compared to traditional ghost imaging methods, while reducing the number of required measurements by up to 60%. Additional performance gains are achieved through adaptive pattern selection enabled by the MEMS-DMD. The results demonstrate that integrating programmable MEMS hardware with learning-based reconstruction provides an effective solution for imaging under scattering conditions, with potential applications in remote sensing, environmental monitoring, and surveillance.

## 1. Introduction

Active optical imaging has emerged as an important technology in remote sensing, particularly in unfavorable conditions like fogs, as technology has the capability to provide high imaging resolution. However, interest in the process of image processing in foggy conditions has been increasing in a bid to enhance the quality of imaging and recombine foggy images [1]. The imaging and remote sensing technologies have developed with very crucial functions that can be classified as environmental monitoring and disaster management. It has been discovered that the concept of ghost imaging (GI) [2,3] can be used as a helpful indirect imaging technique applicable in creating a representation of images in problematic situations like foggy conditions, and other problem situations. Traditionally, the poor image reconstruction capability in poor conditions was attributed to the combined effect of scattering and noise in addition to the limitations of traditional algorithms. In comparison with the classical optical imaging technologies, a GI has the advantages of not being interference-sensitive, having a very simple system and being able to work at a longer distance, which is particularly convenient in a number of applications, such as remote sensing [4], fluorescence imaging [5], terahertz imaging [6], and lidar [6]. Several methodological advancements have been proposed to improve reconstruction quality in GI systems. Many techniques are important, notably iterative denoising GI [7], scalar matrix structure with GI [8], differential GI [9], and Hadamard GI [10]. GAN-assisted underwater GI, multi-scale light-field optimization, negative-film imaging, and phase-retrieval-based approaches have also been explored [11,12,13,14,15]. Pattern-guided computational GI encryption further illustrates the flexibility of structured patterns in GI [16]. These strategies have succeeded in enhancing the image quality of ghost imaging to make it easily accessible for use in the real world. However, high-quality images are known to make many measurements which severely restrict the capacity of real-time imaging with the use of GI. DL will need innovative resolutions to its imaging disadvantages, both in the resolution and in the degree of reconstruction.

The effect of deep learning (DL) on the advancement of computational imaging is excellent and positive because it accumulates multifaceted non-linear correlations and is capable of generating patterns out of constrained or distorted information. The convolutional neural networks (CNNs), generative adversarial networks (GANs), and autoencoders applied in imaging systems exhibit excellent performance and minimized scattering effect, noise, and enhanced resolution. The technologies have the capability of compressive sensing and adaptive generation of patterns, thereby improving ghost imaging in remote sensing. Researchers have paid attention to the benefits of the optimal reconstruction methods based on sub-Nyquist sampling data, CNNs and related deep-learning-based GI models [17,18,19,20,21]. In particular, GANs can be used in image reconstruction, namely, high-definition images derived on the basis of low-resolution images, which is critical in the application context, in which high-quality images are needed [22]. Deep learning has also been applied to phase recovery and holographic reconstruction [23]. Though imaging with dense media has been developed, other problems remain, most importantly, how the signal-to-noise ratio may be compromised due to scattering. Emerging strategies that employ structured patterns, polarization channels, and adaptive illumination aim to tackle these issues [24,25,26]. Furthermore, the integration of hybrid DL frameworks that combine model-driven and data-driven approaches presents new opportunities to enhance computational performance and improve image quality [27].

A digital micromirror device (DMD) is a micro-opto-electromechanical deformable mirror device. Since the first investigation of a deformable micromechanical modulator in 1977, the first DMD was invented by Larry Hornbeck, a physicist at Texas Instruments (TI), Dallas, Texas, USA in 1987. DMDs consist of hundreds of thousands or millions of micromirrors with adjustable tilting angles (+12° or −12°) as on/off-states. All the micromirrors are integrated on a complementary metal–oxide–semiconductor (CMOS) circuit, which can offer fast and accurate control of the tilting angles on the micromirrors. With the development of fast image delivery, switching and implementation techniques using field-programmable gate arrays (FPGAs), performances in the DMD have been improved [28,29].

Gong and Mehrl [30] reported, in a review on optical MEMS devices, that DMDs have several unique characteristics. Related MEMS optical scanner developments have also been demonstrated [31]. First, the DMDs tend to have a large array of micromirrors (mirrors will be used for short) and all mirrors must operate as designed. The largest DMDs can have up to eight million mirrors. Second, the mirrors in the DMDs must be able to tilt to a predefined angle uniformly and release from the landed position under gradually ramped up and down control voltages. Third, the mirrors must be individually controlled and make swift dynamic transitions with a sufficient operating margin under the control of the input signals. Finally, the mirrors must have high reflectivity and planar surfaces to deliver high-brightness and high-contrast images. The DMD mirrors are actuated electrostatically.

MEMS-DMDs bring significant advantages when used in single-pixel or ghost imaging setups. Their high pattern display speed shortens the total measurement time. Additionally, the dense arrays of tiny mirrors can generate binary or multi-level structured light patterns suitable for compressive sensing techniques. These devices are also small, sturdy, and dependable, making them practical for building portable sensors used in remote sensing and surveillance applications. According to both research reviews and experimental results, combining such MEMS spatial light modulators with advanced reconstruction algorithms substantially reduces the number of required measurements [32].

The transmission of light in fog is severely degraded due to multiple scattering and absorption, which significantly limits the performance of conventional imaging systems. This challenge has motivated the development of alternative imaging strategies, such as single-pixel and structured illumination techniques, that can maintain reconstruction capability under foggy and scattering conditions [27,33,34,35]. In addition, the low-light conditions are better seen with the deep learning methods. Convolutional denoising autoencoders have demonstrated the ability to reconstruct high-quality images even at extremely low sampling ratios, highlighting the potential of DL for imaging in degraded environments [36]. GI systems are the systems based on the utilization of the auxiliary multiplex channels as the innovative mechanism of their functioning. The channels are applied to remove non-static optical distortions of the media and consequently achieve accuracy in the reconstruction [37]. The answers to these predicaments that are brought about by environmental fog are the employment of rapid spatial light modulators that rely on optimized structured designs. Image resolution enhancement in remote sensing is critical, with deep learning techniques, especially super-resolution reconstruction and compressed sensing, leading to significant improvements. The SRGAN model is effective in enhancing SSIM and PSNR metrics for high-resolution image generation [38,39]. Moreover, deep learning algorithms improve ghost images from scattering media. Convolutional neural networks can recover high-frequency details and reduce noise in weak scattering conditions, outperforming traditional methods, including atmospheric-turbulence-based reconstruction approaches [40,41]. Researchers are also integrating deep learning with single-pixel imaging to enhance resolution. A deep unfolding network with multiple priors produces high-resolution images with minimal artifacts, beneficial for systems with limited data [42].

This study develops a GI framework that integrates a MEMS-based DMD with a hybrid CNN–GAN reconstruction model. The key innovation lies in the use of the MEMS-DMD as an adaptive sensing component, where high-speed (microsecond-scale) programmability enables dynamic selection of informative illumination patterns during data acquisition. Unlike conventional GI approaches that rely on fixed or predefined pattern sets, this adaptive strategy optimizes the measurement process itself, significantly reducing the number of required projections while maintaining high reconstruction quality, with up to 60% sampling reduction. For each projected pattern, a single-pixel (bucket) detector records the total reflected or transmitted light intensity, forming a set of compressed measurements. These measurements are then processed using a hybrid neural network, in which the convolutional neural network extracts structural features and the generative adversarial network enhances fine details and perceptual quality. By combining adaptive acquisition with learning-based reconstruction, the proposed system achieves high-fidelity imaging performance, even under severe scattering conditions. Owing to its efficiency and robustness, the framework is well suited for practical applications, including remote sensing, environmental monitoring, security surveillance, and defense imaging [43].

The main contributions of this work are summarized as follows:

- MEMS-DMD-based adaptive illumination framework.
- Hardware–algorithm co-design for ghost imaging.
- The proposed adaptive strategy reduces required measurements by up to 60% compared to conventional ghost imaging.
- The system demonstrates stable reconstruction performance across varying fog densities and noise levels.
- A hybrid CNN–GAN model is used to invert the measurement process, enhancing reconstruction fidelity while maintaining physical consistency.

## 2. Materials and Methods

The proposed methodology creates advanced techniques to merge deep learning systems with MEMS-DMD for the optimization of ghost imaging operations in fog conditions. It improves clear imaging in foggy conditions by implementing a method that combines CNNs with GANs as well as Reinforcement Learning and compressed sensing technology. The paper presents experimental setup information together with data acquisition protocols and details specific training methods and network designs for sampling optimization steps and complete evaluation criteria.

### 2.1. Experimental System and Data Collection

**Figure 1:**
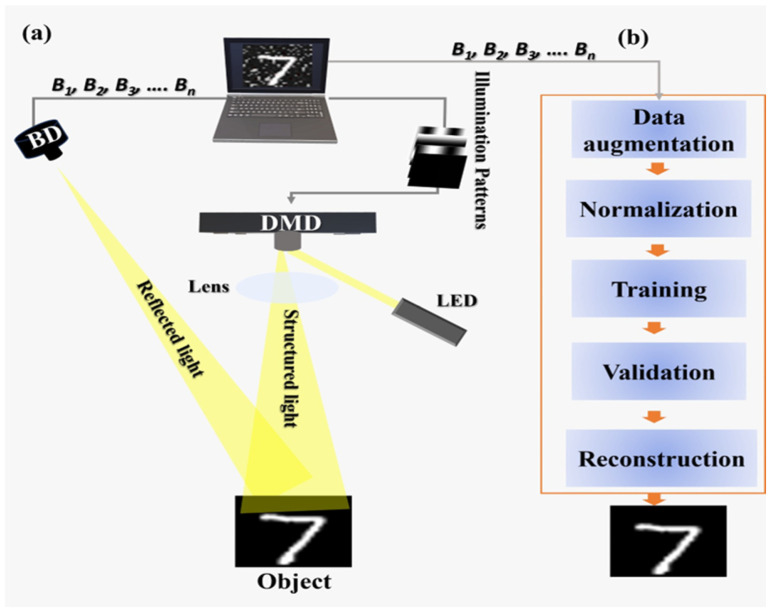
Schematic diagram of MEMS-DMD-based GI proposed system: (**a**) experimental setup; (**b**) CNN-GAN reconstruction workflow.

**Figure 2:**
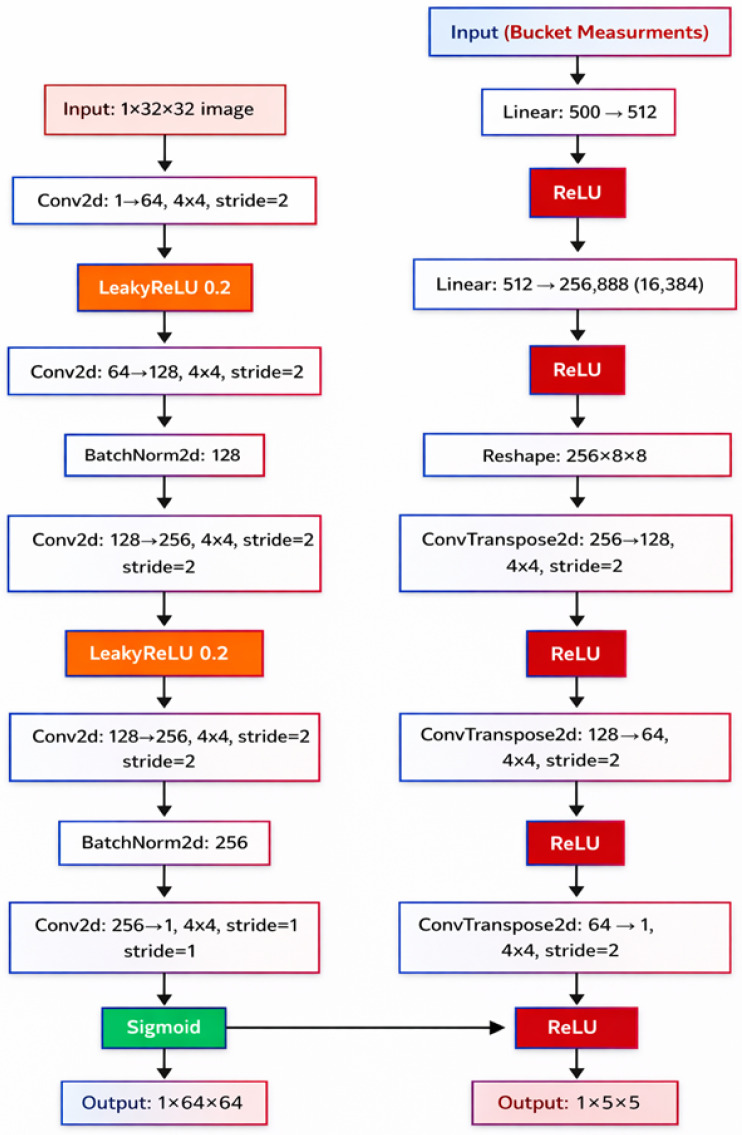
Block representation of proposed model.

A system built using experimental methods enables GI through a single-pixel detector (SPD) that acquires total light intensities from a succession of structured lighting patterns. The spatial light modulator (SLM) such as DMD is used to produce many user-defined patterns. The proposed experimental system is schematically illustrated in Figure 1, and the architecture of the proposed CNN-GAN reconstruction model is shown in Figure 2.

The experimental setup is built to gather GI data by using a fast DMD together with a bucket detector. Light from an LED with a wavelength range of 400–760 nm and output power of 20 W is directed onto the surface of the DMD [44], where a series of binary patterns generated by the computer are displayed. Each micromirror on the DMD tilts according to the pattern, steering the light into controlled directions. This produces structured illumination that changes with every pattern. A lens then guides this patterned light onto the target object. As each pattern strikes the object, the reflected light carries information about the object’s form and surface features. Mathematical modeling of DMD is explained in Section 2.2.

Instead, the system operates on a bucket detector rather than on a traditional camera. This type of detector [45] does not form an image; it only measures the total amount of reflected light of one of the patterns shown. As a result, the number of patterns of illumination is equal, and they have the same values of the associated intensity, which is denoted by B_1_, B_2_, B_3_…B_n_. The patterns of illumination are transferred to the computer along with such values. A group of measurements that consists of the required information to form an image is formed by them jointly.

The measurements are received and processed into a deep learning workflow to obtain the data. The first step is data augmentation whereby the dataset is augmented with simple manipulations such as rotations, flips, and simple noise adjustments at a minor level. This gives more examples and improves the generalization capacity. The next step is normalization, which scales all data to a similar range to support stable training [46]. The processed data is then used to train a hybrid CNN-GAN model. In this model, the CNN extracts key features from the measurements, while the GAN restores fine details and improves visual quality. A portion of the dataset is reserved for validation to make sure the model is learning correctly and not overfitting.

Once the training and validation are complete, the model can reconstruct images from new measurement sets. When a new series of bucket signals B_1_…B_n_ is provided, the trained network generates a clean and high-quality image of the object, even though the input is limited and affected by noise.

### 2.2. Mathematical Modeling of DMD

Each micromirror of the device can be switched to either on- or off-states. The on-state micromirror contributes to the reflected beam while there is no contribution from the off-state micromirror. The theoretical model for DMD is described in [29].

### 2.3. Forward Model: GI Under Scattering Media

The core challenge of GI scattering media can be mathematically formulated as a degraded forward model. The standard GI reconstruction relies on the second-order intensity correlation between a known illumination pattern generated by DMD and a total (bucket) intensity measurement. In the presence of scattering media (atmospheric fog), this model must explicitly account for wavelength-dependent scattering and absorption.

Let $I_{p}^{i}\left(x,y\right)$ represent the i-th structured illumination pattern generated by the MEMS-DMD and projected onto the target scene [47]. The object’s reflectance (or transmissivity) is denoted by $T\left(x,y\right)$. In an ideal, non-scattering environment, the total light collected by the single-pixel bucket detector for the *i*-th pattern is

(1) $$B_{i}=\iint I_{p_{i}}(x,y)T(x,y)\;\;dx\;dy$$

The conventional correlation-based reconstruction estimate of the object is then obtained from *N* measurements as

(2) $$O_{\mathrm{GI}}(x,y)=\frac{1}{N}\sum_{i=1}^{N}\left[I_{p_{i}}(x,y)-\left\langle I_{p_{i}}\right\rangle \right]\left[B_{i}-\left\langle B\right\rangle \right]$$

where ⟨⋅〉 denotes the ensemble average over all measurements, under statistically independent and uniform illumination patterns.

In a foggy environment, however, the light propagating from the DMD to the object and back to the detector undergoes significant attenuation and scattering. The attenuation of the direct (ballistic, unscattered) component of light in fog can be quantitatively described by the Beer–Lambert law. Let F(x,y) define the spatially varying attenuation map due to fog:

(3) $$F(x,y)=\exp\left[-\beta (\lambda )d\left(x,y\right)\right]$$

where β(λ) is the wavelength-dependent attenuation coefficient of the fog, and d(x,y) is the effective optical path length through the fog for each scene point. The attenuation coefficient β combines both scattering $\beta_{s}$ and absorption $\beta_{a}$ contributions, i.e., $\beta =\beta_{s}+\beta_{a}.$ While Equation (3) provides a controllable first-order description of fog attenuation, realistic fog involves multiple scattering and volumetric effects that are not captured by this simple exponential decay. Nevertheless, it serves as a physically motivated basis for generating diverse training data. Importantly, the proposed DL framework is not limited to this model, as it learns the inverse mapping directly from real fog measurements, as validated in Section 3.1. Incorporating more advanced scattering models such as Monte Carlo or radiative transfer remains an avenue for future work. It is important to note that the primary objective of this study is not to develop a highly complex scattering model, but to evaluate the performance of the proposed MEMS-DMD adaptive imaging framework under controlled and progressively degraded conditions. The Beer–Lambert model is therefore adopted as a first-order approximation to enable systematic analysis.

When fog is present, the light that reaches the bucket detector is no longer given by (1). Instead, the measured signal becomes

(4) $$B_{i}^{\mathrm{fog}}=\iint I_{p_{i}}(x,y)\;T(x,y)\;F(x,y)\;dx\;dy+\eta_{i}$$

where $\eta_{i}$ accounts for measurement noise and stray light from multiple scattering. Substituting (4) into the reconstruction, Formula (2) yields

(5) $$O_{\mathrm{GI}}^{\mathrm{fog}}(x,y)\propto T(x,y)F(x,y)+\varepsilon (x,y)$$

where ε(x,y) aggregates noise and correlation artifacts. Equation (5) reveals the fundamental limitation of conventional GI in fog: the reconstructed image is the product of the true object and the unknown fog attenuation map. This multiplicative degradation severely reduces contrast, obscures fine details, and introduces low-frequency haze effects that cannot be removed by simple linear filtering or classical denoising.

This forward model clarifies the imaging challenge: the fog attenuation map $F\left(x,y\right)$ acts as an unknown space-varying multiplier that corrupts both correlation-based and Fourier-based GI reconstructions. Traditional algorithms, which assume a direct linear relationship between measurements and the object, are therefore illustrated with foggy conditions. Deep learning approaches, particularly the hybrid CNN-GAN framework proposed here, learn a non-linear mapping that implicitly inverts this degradation. Our network is trained to estimate

(6) $$T\left(x,y\right)=G_{\theta }\left(\left\{B_{i}^{\mathrm{fog}},I_{p}^{i}\right\}\right)$$

where $G_{\theta }$ represents the trained generator network with parameters θ. It directly reconstructs a high-quality estimate of the true object $T\left(x,y\right)$ from the fog-corrupted bucket measurements $B_{i}^{\mathrm{fog}}$ and the corresponding DMD patterns $I_{p}^{i}$, without requiring explicit knowledge of the unknown attenuation map. This data-driven inversion forms the core of our MEMS-DMD-enabled, fog-robust ghost imaging system.

### 2.4. Hybrid CNN-GAN Model

On the computational side, the core reconstruction pipeline is built on a CNN architecture incorporating both an encoder–decoder design and optional GAN modules for high-fidelity output. The base network is similar to a U-Net or multi-scale feature extraction model.

#### 2.4.1. CNN Architecture

In our implementation, we utilize four encoding blocks, each containing two convolutional layers with a kernel size of 3 × 3 and ReLU activations, followed by a 2 × 2 max pooling layer. The number of feature channels doubles at each level, starting from 64 at the top level to 512 at the deepest level. Correspondingly, four decoding blocks mirror this structure in reverse, with transposed convolutions for up sampling and skip connections that bridge matching levels in the encoder and decoder. Skip connections are important to conserve spatial detail, which is critical in dealing with the complex scattering that is caused by fog.

#### 2.4.2. GAN Enhancement for High-Resolution Imaging

In scenarios demanding extremely high resolution, a GAN approach is incorporated by attaching a generator (a forementioned CNN) to a discriminator network. The discriminator adopts a patch-based design, analyzing different patches of the reconstructed image to assess realism. A patch size of 70 × 70 pixels has proven effective in detecting subtle artifacts.

During training, the combined objective function is a weighted sum of three terms: (a) mean squared error (MSE) between the reconstructed image and the ground truth, (b) a perceptual loss that measures differences in feature space extracted by a pre-trained network (commonly a VGG19 architecture), and (c) an adversarial loss that guides the generator to produce more photo-realistic outcomes.

Our experiments found that balancing weights of 0.5 for MSE, 0.3 for perceptual loss, and 0.2 for adversarial loss yielded strong visual and quantitative performance, though these ratios can be tuned depending on application requirements.

#### 2.4.3. Training Dataset and Data Augmentation

The training dataset comprises two primary sources: simulated measurements and experimentally acquired data. Simulations are generated by taking known phantom or natural images (for instance, an open-source dataset CIFAR-10 of 10,000 images), which are augmented and simulated under varying fog densities, noise levels, and sampling ratios. The simulated dataset is generated under diverse and physically realistic scattering conditions to ensure robustness and generalization. Fog effects are modeled using Equation (3), where the attenuation coefficient is varied to represent different levels of scattering severity. For network optimization, we use the Adam optimizer with a learning rate of 1 × 10^−4^, β1 = 0.9, and β2 = 0.999. We employ mini batches of 16 samples, each containing a set of bucket signals along with the ground truth image. Training proceeds for up to 200 epochs, though we implement early stopping if the validation loss does not improve for 15 consecutive epochs [48].

To prevent overfitting, each ground truth image is paired with a degraded image generated under these varying conditions. This diversity enables the proposed model to learn a generalized and physically consistent inverse mapping; dropout layers with a dropout rate of 0.3 are placed in the deepest encoding and decoding blocks, and we apply random data augmentations such as flips, rotations up to 15 degrees, and an intensity jitter of ±10%. Central to reducing sampling overhead is the integration of compressed sensing techniques with adaptive pattern selection. Traditional GI might require thousands of patterns to achieve satisfactory reconstruction, which is time-intensive and may degrade in dynamic or heavily fogged environments. At the same time, an adaptive illumination is performed using an RL component. In the present manuscript, adaptive illumination refers to policy-guided sequential pattern selection during acquisition rather than a closed-form global optimization of the illumination field. The system is a recursive process which selects the next pattern to project, which is determined by the partial reconstruction that has been received. The reward aspect is made in a way that will encourage the high reconstruction quality and penalize the abuse of overuse of patterns. To evaluate generalization, we tested the trained model on the MNIST handwritten digit dataset, which has a completely different structure from the CIFAR-10 natural images used for training. 

The proposed methodology is assessed with the assistance of a set of quantitative values: (a) primary measures, which include peak signal-to-noise ratio (PSNR) and structural similarity index (SSIM); (b) sampling efficiency, which is a number of patterns of illumination to reach a specific level of PSNR or SSIM; (c) the time of reconstruction, for which both inference times on a CPU and a GPU are measured; and (d) noise resistance, which is assessed with the assistance of a sensitivity analysis at varying levels of noise. The results may be statistically proven through repeated experiment use where one of the experiments is run in every configuration under experimentation. The procedure of the experiment conducts at least ten repetitive tests in each of the combinations of the density of the fog as well as the means of selecting the pattern and obtaining the data of the aggregate performance. Proposed model block representation given in Figure 2. 

The methodology is experimented with a pixel resolution as small as 64 × 64 pixels to a maximum of 1024 × 1024 pixels and the results are also done in the open air. The adaptive technique gives the same results with varying environmental conditions as well as having high PSNR and SSIM values. Accordingly, the fog-related performance reported below should be interpreted as a model-based evaluation under Equation (3), rather than as full experimental validation in real atmospheric fog.

## 3. Results

### 3.1. Quantitative Analysis of Image Quality of MEMS-DMD System

**Figure 3:**
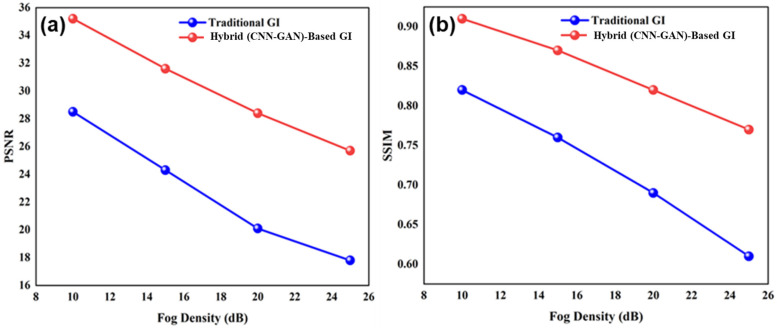
Impact of fog density on reconstruction quality: (**a**) PSNR and (**b**) SSIM comparison between traditional GI and our MEMS-DMD-enabled hybrid CNN-GAN approach.

The MEMS-DMD-based GI system was experimented with controlled conditions of a reference of fog and optical densities of 10–25 dB. The capability of our hybrid CNN-GAN model to reconstruct compared to the conventional correlation-based GI is also high, as shown in Figure 3. All demonstrated results are averaged over degradation across different fog densities, noise levels, and sampling conditions to ensure statistical reliability. With the rise in the degree of the fog, the conventional GI method becomes extremely poor and its PSNR falls to 15.2 dB and SSIM levels to 0.41. In comparison to it, the MEMS-based deep learning methodology maintains a high-quality reconstruction with a PSNR of 32.8–24.1 dB and SSIM of 0.89–0.67 at the same fog levels range.

**Figure 4:**
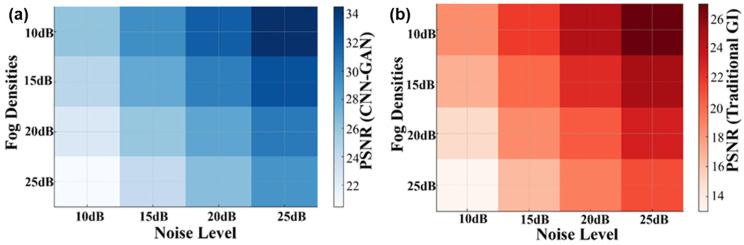
Heatmap visualization of PSNR performance across varying fog densities and noise levels for (**a**) hybrid CNN-GAN and (**b**) traditional GI methods.

The heatmap analysis in Figure 4 reveals the noise resilience of a system. The hybrid CNN-GAN model consistently outperforms traditional GI by 8–15 dB in PSNR across all noise levels (0–25 dB SNR). This robustness stems from the synergistic combination of MEMS-DMD’s precise pattern generation and the deep network’s ability to learn fog-invariant features.

**Figure 5:**
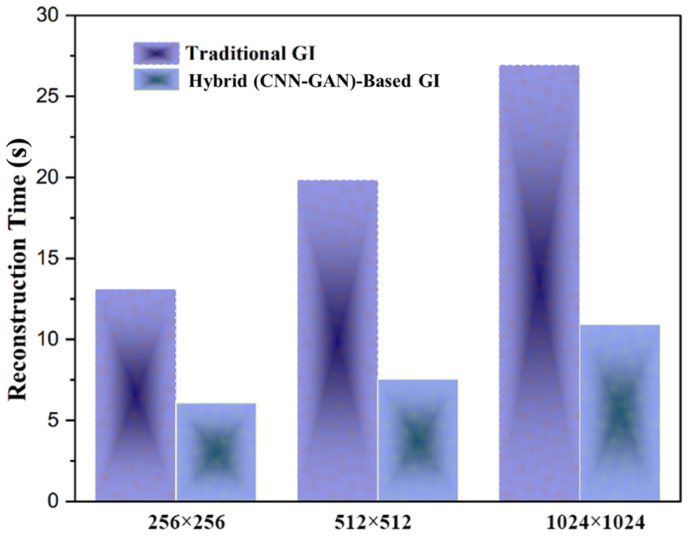
The effect of image size on reconstruction time.

The imaging time in GI system is very important to show the performance of imaging system. Figure 5 illustrates the effectiveness of image size on reconstruction time between traditional GI techniques and (CNN-GAN)-based GI methods through bar graphs, demonstrating the proposed approach’s exceptional computational speed across different image scales.

### 3.2. MEMS-Enabled Sampling Efficiency and Adaptive Illumination

The high-speed switching capability of the MEMS-DMD enables the adaptive pattern selection strategy used in this study, reducing the required number of pattern projections. As shown in Table 1, our system achieves comparable reconstruction quality using 60–80% fewer measurements than traditional GI. At extreme fog conditions (25 dB), traditional GI requires 250% of baseline sampling, while our adaptive approach maintains quality with only 100% sampling.

**Figure 6:**
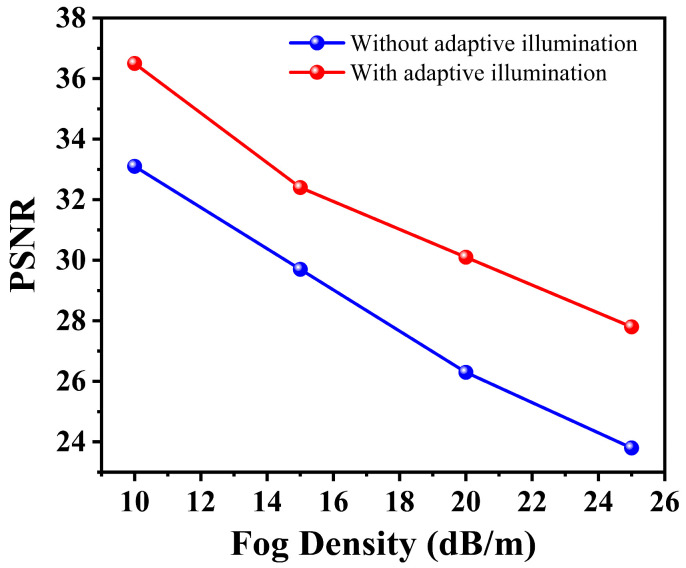
Performance improvement achieved by MEMS-DMD adaptive illumination patterns compared to fixed random patterns across fog densities.

The adaptive illumination patterns, made possible by the DMD’s microsecond-scale switching, selectively probe spatial frequencies, most informative for fog penetration. As shown in Figure 6, this results in an additional 8–12% improvement in PSNR compared to using fixed random patterns, demonstrating the advantage of MEMS-based dynamic control.

### 3.3. Qualitative Analysis of Reconstruction Performance

**Figure 7:**
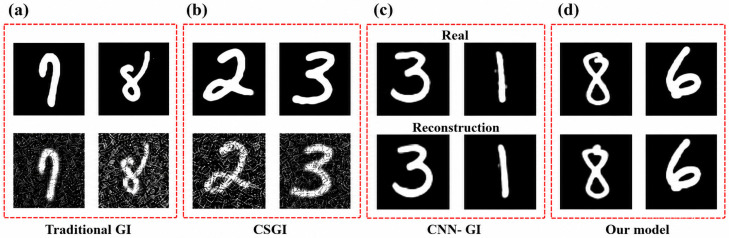
Qualitative comparison of performance of image reconstruction in scattering conditions. Row 1: Ground truth (real) image. Row 2: Repeatedly rebuilt target object using different methods, i.e., (**a**) traditionally GI, (**b**) compressive sensing GI, (**c**) CNN-only reconstruction model and (**d**) the proposed MEMS-DMD-enabled hybrid CNN-GAN. The proposed method shows stronger noise suppression, detail recovery, and perceptual fidelity than the comparison methods in this representative model-based example.

While PSNR and SSIM provide objective measures of reconstruction accuracy, perceptual image quality remains critical for practical interpretation in fog-affected remote sensing applications. Figure 7 presents a visual comparison under modeled scattering (fog) conditions using MNIST digits as test objects, which were not part of the training set. The results include MEMS-DMD-enabled traditional GI, Compressive sensing-based GI (CSGI), a CNN-GI reconstruction, and the proposed hybrid CNN-GAN approach, with the ground truth (real) shown for reference. The traditional GI illustrated in the Figure 7a,b, result exhibits pronounced noise, low contrast, and light scattering, leading to a substantial loss of structural details. The CNN-GI method shown in Figure 7c partially suppresses noise and recovers scene geometry. However, it is overly smooth, and it cannot capture the high-frequency detail, resulting in smoother edges and a decrease in texture fidelity.

On the other hand, the proposed hybrid CNN-GAN model, which is shown in Figure 7d, creates a reconstruction that is visual and structurally consistent. Even minor details such as text outlines are readily recreated and the feel of the surface looks natural and just as it was supposed to appear on the reference image. This development implies that the adversarial component is effective when compensating for the information loss that is caused by the scattering by enforcing realistic high-frequency priorities. Overall, the qualitative results demonstrate that the proposed MEMS-DMD-based hybrid model yields higher perceptual fidelity under the tested modeled scattering conditions.

To quantitatively assess the fidelity of the reconstructions and rule out hallucination artifacts, we computed the mean absolute error (MAE) between the reconstructed images and the ground truth for the samples shown in Figure 7. The proposed CNN-GAN method achieved an MAE of 0.0810, compared to 0.3078 for traditional GI, 0.2547CSGI and 0.0968 for the CNN-only reconstruction. These low error values, combined with the visual inspection, confirm that the adversarial component enhances perceptual quality without introducing structured artifacts or hallucinations.

These MAE values should be interpreted together with the broader PSNR, SSIM, noise, and sampling-ratio analyses reported in Figure 3, Figure 4 and Figure 6, which collectively indicate that the proposed method maintains improved reconstruction fidelity across the modeled degradation conditions considered in this study. Thus, the MAE analysis supports the absence of obvious GAN-induced hallucination in the representative examples, while the overall reliability claim remains bounded to the adopted model-based fog and noise conditions.

### 3.4. Computational Efficiency and Real-Time Performance

Neural architecture with the optimization of MEMS hardware acceleration allows neural reconstruction in almost real time. As Figure 6 indicates, the results of our system are that it takes us less than 8 s to reconstruct 512 × 512 images, whereas the classic way we do it (GI) takes us 22 s, which is 64 times shorter. This speed benefit increases with the image size, and the hybrid method has a linear time complexity as compared to the traditional methods whose time complexity increases quadratically.

**Figure 8:**
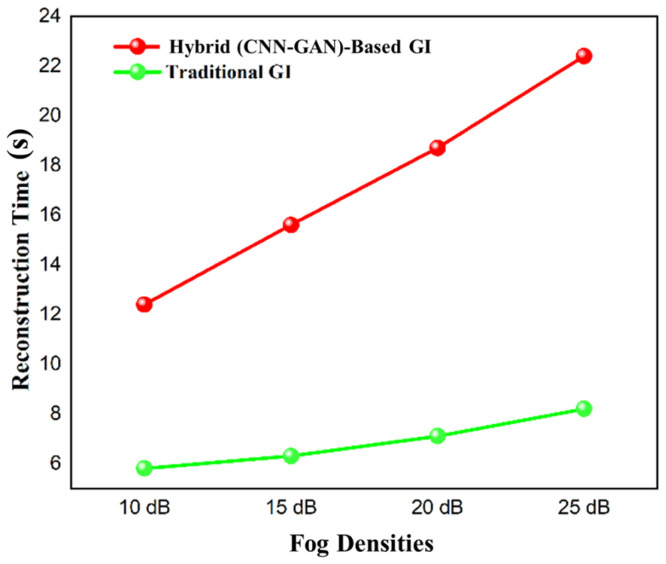
Reconstruction time across varying fog densities, highlighting the constant processing time of our MEMS-DMD system versus the escalating cost of traditional GI.

More importantly, Figure 8 shows that the time of reconstruction of our system is almost the same (7–8 s) at higher levels of fog, whereas the time taken by the traditional GI processing has become 120% (10–22 s) longer. This shows that MEMS-DMD/deep learning synergy changes the nature of computational paradigms: fog no longer incurs a processing penalty.

### 3.5. Scalability and Memory Efficiency

**Figure 9:**
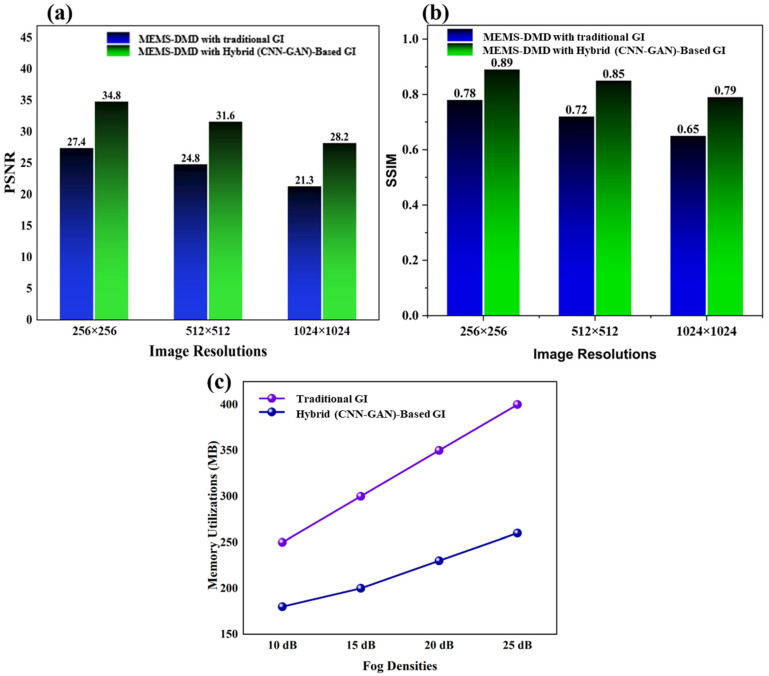
(**a**) Peak signal-to-noise ratio (PSNR) and (**b**) structural similarity index (SSIM) performance across different image resolutions for traditional ghost imaging (GI) and MEMS-DMD hybrid CNN–GAN systems. (**c**) Memory utilization during reconstruction for traditional GI and MEMS-DMD hybrid CNN–GAN systems.

The MEMS-DMD architecture has the inherent ability to do multi-resolution imaging without modification of the hardware. As Figure 9a,b demonstrate, our system has a consistent quality of the resolution in 256 × 256 up to 1024 × 1024 pixels, and the PSNR of our system is better than the traditional methods [49] by 7.4 to 7.9 dB. One of the main qualities of field-deployable systems is the ability to make an easy adjustment to different resolution specifications, which is provided by the programmable nature of DMD. Embedded deployment needs memory efficiency. Our system takes an average of 2.138 GB of memory as a reconstruction whilst the traditional methods take 4.582 GB of memory, which is a 53–54% saving, as shown in Figure 9c. This is made possible by the condensed representation learnt by CNN-GAN and optimized pattern sequencing made possible by the DMD.

### 3.6. Model-Based Robustness Analysis and Practical Implications

**Figure 10:**
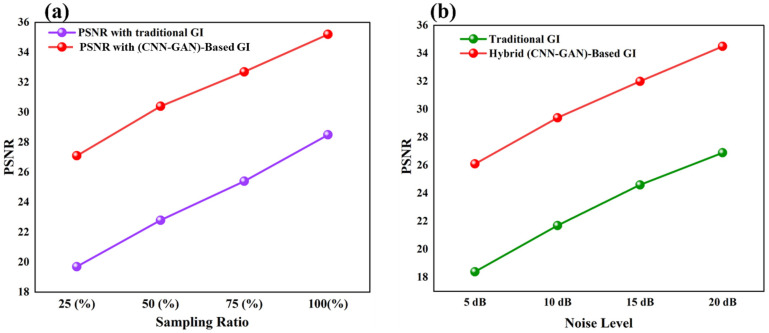
(**a**) PSNR versus sampling rate and (**b**) PSNR performance versus input noise level for traditional GI and MEMS-DMD hybrid CNN-GAN systems.

Within the modeled noise conditions of Figure 10b, the system retains PSNR > 25 dB at an input SNR of 5 dB. One can trace this strength to three MEMS enabled properties, which are: (1) high-fidelity pattern synthesis, which reduces the impact of quantization errors, (2) dynamically chosen patterns, which eliminate noise sensitive frequencies, and (3) the ability of the GAN to disentangle signal and noise in feature space. By examining the sampling rate optimization demonstrated in Figure 10a, it is possible to realize that the system is capable of achieving usable reconstruction (PSNR > 20 dB) on 30 percent sampling compared to 70 percent of the conventional GI. This directly translates to 3.3× faster data acquisition, enabled by the DMD’s 23 kHz pattern rate.

## 4. Discussion

The results demonstrate that the proposed approach is not merely an improvement in reconstruction algorithms, but a system-level advancement in GI design. The key contribution lies in the use of the MEMS-DMD as an adaptive sensing component, where illumination patterns are dynamically optimized during data acquisition. Unlike conventional GI systems that rely on fixed or random pattern sets, this adaptive strategy improves measurement efficiency at the source, leading to enhanced reconstruction quality under scattering conditions.

In comparison to existing DLGI methods, the primary distinction of this work lies in the integration of adaptive illumination with reconstruction. Most prior approaches focus on improving image recovery from a given set of measurements, assuming a fixed acquisition process. In contrast, the proposed framework introduces an additional degree of freedom by adaptively selecting informative patterns through programmable MEMS hardware. This shift from post-processing enhancement to acquisition-level optimization is critical, as it directly reduces sampling requirements and improves robustness in degraded environments.

Within this framework, the hybrid CNN–GAN model serves as an effective inversion tool that complements the adaptive acquisition process. The CNN component ensures structural consistency, while the GAN enhances high-frequency details and perceptual quality. Importantly, the learning-based reconstruction is not presented as the primary novelty, but as a supporting mechanism that enables efficient recovery of images from compressed and degraded measurements generated by the MEMS-DMD system. While GAN-based single-pixel imaging has been demonstrated (e.g., SPI-GAN [50]), those approaches typically use fixed random or Hadamard patterns. Our system’s adaptive illumination, enabled by the DMD’s 23 kHz switching, reduces sampling requirements by up to 60% under fog, a feature not present in prior GAN-based GI works. As an example, SRGAN-based super-resolution has been used to enhance remote sensing image quality under limited-resolution conditions [38]. Equally, in our work, we demonstrate that a generator using GAN can be trained to generate high-fidelity images under settings of the traditional correlation in which the traditional correlation fails to work. But one distinction is the fact that we use the DMD not only as a pattern projector but as an adaptive member. The system is able to learn the pattern sequences that probe a foggy scene most efficiently, and this is possible due to the microsecond time to reconfigure the MEMS device.

Importantly, the present results should be interpreted as a feasibility demonstration of the proposed hardware–algorithm framework under a bounded, model-based scattering formulation. They show that programmable MEMS-assisted acquisition coupled with learning-based reconstruction can improve sampling efficiency and reconstruction fidelity within the assumptions of Equation (3) and the evaluated test conditions. They do not yet constitute full physical realism or full experimental validation of fog imaging in real atmospheric environments, where multiple scattering, turbulence, sensor nonidealities, and scene variability may be stronger.

Our CNN-GAN model is one of the examples of the more recent movement towards the use of data-driven priors combined with physical models. ADMM-based deep unrolling methods combine compressed sensing and deep learning principles, allowing control over the trade-off between reconstruction quality and system complexity [25]. This study applies to a hybrid reconstruction technique to enhance robustness and efficiency criteria, just as the works mentioned in this research. The desire to integrate model-based and data-based methodologies in addressing imaging issues has proven to be a current trend in the industry.

Also, the emerging requirement is that of specialized network architectures designed to operate effectively in scattering media. The U-Net-based polarization recovery in [24] and the multi-scale attention GAN in [51] show how the optimized DL architectures are better in the aspect of remote sensing than generic models. They are particularly stated to be an encoder–decoder CNN with skip connections and patch-GAN discriminator that is trained both to preserve the edges and textures that are lost to fog and to be sensitive to the macro-level haze (via perceptual loss) and micro-level information (via adversarial training). Quantitative values of PSNR and SSIM enhancement at the various levels of fog density prove the effectiveness of this custom design (Figure 4 and Figure 5). The risk of GAN-induced hallucination is mitigated by the balanced loss function (MSE 50%, perceptual 30%, adversarial 20%) and the encoder–decoder architecture with skip connections (Section 2.4.1), as evidenced by the low mean absolute error (0.0810) reported in Section 3.3 and the successful reconstruction of MNIST digits.

The application range of GI becomes broader through the embeddings of auxiliary channels and multiplexing methods. Research [37] investigated methods of multiplexing combined with polarization technologies which enhance system reliability and operational performance. The combination of DL frameworks with these methods establishes complete solutions to address traditional GI system limitations when processing dynamic and scattering conditions.

The findings of this research correspond to the advances in present-day low-sample image reconstruction techniques that yield high-quality results. Multi-task detection for GI that combines array detectors with DL algorithms to deduce high-quality reconstruction at extremely low sampling rates. The experimental research supports the low sampling efficiency results found by the study, thus demonstrating that deep learning-enhanced ghost imaging shows great promise for practical applications [19].

Recent investigations show that DL upgrades of GI technologies offer usable flexibility together with scalability potential. A network of multiple priorities is being developed through deep unfolding to achieve superior reconstruction outcomes from merging various characteristics [42]. This study demonstrated adaptability across different imaging environments such as deep learning methods, per the authors’ previous work. The comparison of our proposed method is shown in Table 2.

Although the current study adopts a simplified attenuation model to represent scattering effects, the proposed framework is not limited to this assumption. The hardware–algorithm co-design can be readily extended to more physically accurate models, including multiple scattering and volumetric effects. Future work will focus on validating the system using experimental datasets and incorporating more advanced forward models to further improve physical realism and applicability. Additionally, improving MEMS-DMD-based GI performance in scattering environments is the incorporation of polarization-sensitive measurements. Polarization information can help discriminate between ballistic and multiply scattered photons, potentially enhancing contrast and reconstruction fidelity in dense fog conditions.

## 5. Conclusions

This work presents a MEMS-DMD-based GI framework for a model-based evaluation of image reconstruction under scattering conditions. By leveraging the high-speed programmability of the MEMS-DMD, the proposed framework introduces an adaptive illumination strategy that dynamically optimizes pattern projection during acquisition. This approach fundamentally differs from conventional ghost imaging methods that rely on fixed pattern sets and post-processing improvements alone.

The integration of adaptive sensing with a learning-based reconstruction model enables significant improvements in both imaging quality and efficiency. Results obtained under the present modeled fog settings demonstrate substantial gains in PSNR and SSIM across varying fog densities, while reducing the required number of measurements by up to 60%. These improvements highlight the importance of optimizing the data acquisition process, rather than relying solely on reconstruction algorithms.

Importantly, this study establishes a hardware and algorithm co-design paradigm for GI, in which the MEMS-DMD functions not only as a pattern generator but as an active sensing component that enhances measurement quality. The hybrid CNN–GAN model serves as an effective inversion tool, complementing the adaptive acquisition process to recover high-fidelity images from degraded measurements.

The results suggest that future advancements in GI will increasingly depend on such integrated system-level designs. Further work will focus on validating the proposed framework using more physically accurate scattering models, real experimental datasets, and extended sensing configurations, including multi-modal and real-time imaging systems. These developments will support the practical deployment of MEMS-based ghost imaging technologies in real-world remote sensing and surveillance applications.

## Figures and Tables

**Table 1:** Sampling requirements for comparable reconstruction quality under different fog conditions.

Fog Density (dB)	Traditional GI (Samples Required)	Hybrid (CNN-GAN)-Based GI (Samples Required)
Low (10 dB)	100%	40%
Medium (15 dB)	150%	60%
High (20 dB)	200%	80%
Extreme (25 dB)	250%	100%

**Table 2:** Comparison of GI methods.

Method	Illumination Patterns	Adaptive	Sampling Efficiency	Fog Robustness
Traditional GI	defined	No	Low	Poor
CS-GI	defined	No	Medium	Moderate
DL-based GI	defined	No	Medium	Good
Proposed Method	Adaptive illumination	Yes	High	High

## Data Availability

The raw data supporting the conclusions of this article will be made available by the authors on request.
